# A contemporary risk model for predicting 30-day mortality following percutaneous coronary intervention in England and Wales

**DOI:** 10.1016/j.ijcard.2016.02.085

**Published:** 2016-05-01

**Authors:** Katherine S.L. McAllister, Peter F. Ludman, William Hulme, Mark A. de Belder, Rodney Stables, Saqib Chowdhary, Mamas A. Mamas, Matthew Sperrin, Iain E. Buchan, On behalf of the British Cardiovascular Intervention Society and the National Institute for Cardiovascular Outcomes Research

**Affiliations:** a Health eResearch Centre, Farr Institute, University of Manchester, UK; b Queen Elizabeth Hospital, Birmingham, UK; c The James Cook University Hospital, Middlesbrough, UK; d Institute of Cardiovascular Medicine and Science, Liverpool Heart and Chest Hospital, Thomas Drive, Liverpool, UK; e Keele University, UK; f Manchester Heart Centre, Central Manchester NHS Foundation Trust, Manchester, UK

**Keywords:** AUC, area under the receiver operating characteristic curve, BCIS, British Cardiovascular Intervention Society, LVEF, left ventricular ejection fraction, NHS, (UK) National Health Service, MI, myocardial infarction, NICOR, National Institute for Cardiovascular Outcomes Research, NWQIP, North West Quality Improvement Programme, PCI, percutaneous coronary intervention, Angioplasty, Catheterization, Coronary disease, Prognosis, Risk factors

## Abstract

**Background:**

The current risk model for percutaneous coronary intervention (PCI) in the UK is based on outcomes of patients treated in a different era of interventional cardiology. This study aimed to create a new model, based on a contemporary cohort of PCI treated patients, which would: predict 30 day mortality; provide good discrimination; and be well calibrated across a broad risk-spectrum.

**Methods and results:**

The model was derived from a training dataset of 336,433 PCI cases carried out between 2007 and 2011 in England and Wales, with 30 day mortality provided by record linkage. Candidate variables were selected on the basis of clinical consensus and data quality. Procedures in 2012 were used to perform temporal validation of the model. The strongest predictors of 30-day mortality were: cardiogenic shock; dialysis; and the indication for PCI and the degree of urgency with which it was performed. The model had an area under the receiver operator characteristic curve of 0.85 on the training data and 0.86 on validation. Calibration plots indicated a good model fit on development which was maintained on validation.

**Conclusion:**

We have created a contemporary model for PCI that encompasses a range of clinical risk, from stable elective PCI to emergency primary PCI and cardiogenic shock. The model is easy to apply and based on data reported in national registries. It has a high degree of discrimination and is well calibrated across the risk spectrum. The examination of key outcomes in PCI audit can be improved with this risk-adjusted model.

## 1 Introduction

In the UK National Health Service (NHS), efforts to improve the outcomes of coronary revascularisation have received coordinated attention since March 2000 [1]. More recently, the outcomes for units, and now clinicians, have been published —starting with ten surgical domains as part of the “candour” agenda of opening up NHS performance data to public scrutiny [2].

The British Cardiovascular Intervention Society (BCIS, www.bcis.org.uk) is the professional body representing all those involved in the field of interventional cardiology. Since 2005, BCIS has incorporated patient-level data in its long running annual audit of all percutaneous coronary intervention (PCI) procedures performed in the UK. This audit is used for benchmarking performance to help improve services and underpin clinical governance [3]. Due to wide variations in case mix between both operators and PCI centres, crude mortality metrics cannot be used to compare clinical and procedural outcomes. Using index cases to compare outcomes for patients with more homogenous clinical features has several limitations. The preferred approach is to use risk-adjustment techniques that take into account the variability of expected outcomes for patients who present with different combinations of risk factors [4].

The North West Quality Improvement Programme (NWQIP) model has been used for risk-adjusted outcome surveillance since 2006 [5]. This model was developed from data on patients treated in North West of England between 2001 and 2003. Since then there have been major changes in PCI techniques, adjunctive therapies, and in clinical indications for PCI. In 2003 the mainstay of treatment for patients with ST elevation myocardial infarction (MI) was thrombolysis, but by 2012, over 93% of patients were treated with primary PCI [6]. The NWQIP model has been used over this time to adjust for case-mix when auditing the outcomes of PCI. Since the NWQIP model was developed, more than a decade ago, there have been changes in case-mix and clinical practise, most significantly the systematic uptake of primary PCI. Evaluation in a contemporary cohort from the BCIS dataset suggests that while the model retains reasonable overall discrimination for major adverse cerebrovascular or cardiovascular events (MACCEs) it has been subject to significant calibration drift with consistent over-prediction of risk (see Supplementary materials). This inaccuracy demands a new model based on contemporary data. For UK national audit purposes both the data and models need quality assurance. The quality of current adverse event reporting depends on local practises at PCI centres. In this regard, in spite of a series of internal validation checks on data consistency, there are substantial variations in the quality of the audit data returned by different centres [7].

The aim of this study was to produce an updated robust risk adjustment model with good discrimination and correct calibration for contemporary PCI practise in the UK. A similar updating exercise has recently been undertaken in the National Cardiovascular Data Registry in the US [8]. We chose to assess 30 day mortality rather than in-hospital major adverse cerebrovascular or cardiovascular event because the former can be derived from linkage with the Office for National Statistics records (a consistent end point that is not influenced by local variation in data completeness). We excluded patients suffering a cardiac arrest and being treated outside of hospital prior to PCI because this group contains a heterogeneous combination of patients with different risk profiles, and also because of concerns that including such patients might lead to inappropriately risk-averse clinical behaviour [9].

## 2 Material and methods

### 2.1 Definition of dataset and pre-processing

The BCIS database comprises 113 variables describing baseline demographics, clinical presentation, procedural details and outcomes to hospital discharge. Data for all procedures performed in the UK are collected at each PCI centre, encrypted and then uploaded to servers now hosted by the National Institute for Clinical Outcomes Research (NICOR) based at University College London [3]. The Office for National Statistics provides reliable independent tracking of mortality (for patients living in England and Wales only), using linkage by each patient's unique identifier. Cases in Scotland and Northern Ireland were therefore excluded from the model development. Linkage was carried out by the Medical Research Information Service on behalf of NICOR. Analysis was conducted at the University of Manchester with Local Research Ethics Committee approval (reference no.11/NW/0694). The data were cleaned and analysed using Stata® MP v11.2 (StataCorp LP).

**Fig. 1:**
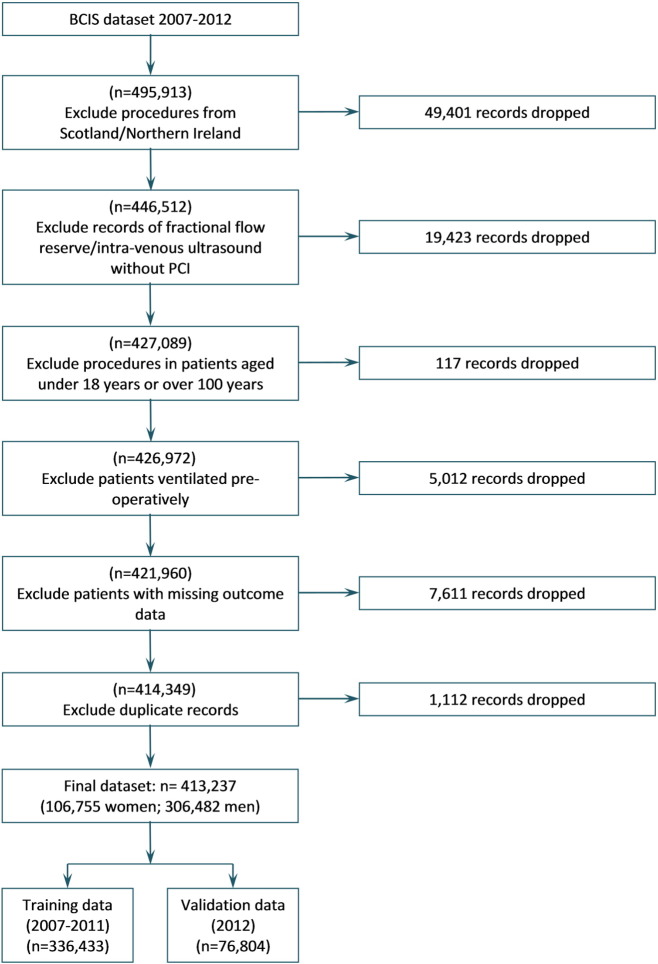
Flow chart illustrating creation of analysis and validation dataset from the available records in the BCIS database.

Although there is no independent validation of data entry, a number of range checks and assessments of internal validity are applied as data are uploaded to NICOR. We performed a sequence of further procedures to clean the dataset. A number of exclusion criteria were applied (Fig. 1). We limited our analysis to patients aged over 18 and under 100 at the time of procedure. Patients outside of these age limits are small in number, but could contribute disproportionately to outcomes. Patients without tracked mortality data were also excluded (this excluded group incorporating all patients from Scotland and Northern Ireland). Patients who were ventilated before PCI were also excluded, this field being used as a proxy indicator for out of hospital cardiac arrest.

A total of 1112 procedures were identified as likely duplicate entries and were also excluded. These were identified by comparing records across age, gender, pseudonymised hospital identifier, pseudonymised patient identifier, pseudonymised date of operation, month of operation, time of operation, urgency, clinical indication for procedure and status at discharge. Data used for the final model are available from the authors where the requester has sought permission from NICOR.

### 2.2 Variable selection and definition

Of the available fields in the BCIS dataset, a shortlist of 10 candidate risk factors was identified by the authors on the basis of clinical consensus and data quality. As the model was intended to be used to predict outcome before the start of a procedure, variables relating to decisions or events occurring during or after the procedure were excluded.

Age at procedure was given in years and months. For modelling, age was mean-centred within the development cohort (mean = 64.8 years); a quadratic age term was also explored. Diabetes was defined as present whether patients were diet controlled, or treated with medication including insulin. Serum creatinine levels were only recently added to the dataset and were therefore missing in earlier years of the development cohort. However, a binary variable indicating whether creatinine measures were greater than 200 μmol/l was available throughout the time period, so this was used as the measure of renal function. Use of dialysis for acute or chronic renal failure was also recorded, and if both this and a creatinine measure of > 200 μmol/l were present, the patient was assigned to the ‘dialysis’ group. Patients with functioning transplants were grouped with those who had no renal impairment, unless on dialysis or with a creatinine > 200 μmol/l.

Definitions of the fields are available online (www.ucl.ac.uk/nicor/audits/adultpercutaneous/datasets). Clinical indication for PCI procedure is recorded as one of 12 possible options in the database. For the purposes of this model we derived a simpler five-group classification to combine the clinical indication and the urgency of the procedure, to avoid the problem of collinearity between these two variables. These groups are described in Table 1.

There were insufficient data available on ethnicity of patients to consider this as a variable in the model. We did not include a measure of left ventricular ejection fraction as data on this characteristic were missing in 50.7% of all patients, and in 67.4% of emergency or salvage patients. Furthermore, not only is LV function rarely known at the time of emergency PCI for STEMI, but also can be labile following intervention. Sensitivity analysis was conducted to evaluate alternative modelling strategies which would enable the inclusion of this risk factor; firstly in a model trained only on cases where data on this risk factor were available, and secondly on a fully multiply-imputed training dataset.

### 2.3 Missing data handling

The percentage of data missing in the shortlisted variables is shown in Table 2. Before excluding patients aged over 100, in cases where age at procedure was recorded as greater than 120 years this was assumed to be erroneous and re-coded as missing. Missing age values in the development cohort were replaced with the median by gender within that cohort (males 63.6 years, females, 69.6 years). The same values were used to replace missing age values in the training cohort, as it was assumed that during model use the median population ages might not be available. For categorical variables, missing values were assigned to the baseline category i.e. it was assumed that if a risk factor was not recorded then it was absent. This represents a plausible missing not at random mechanism that is likely to operate in this case (multiple imputation assumes that data are missing at random), and incentivises improved data collection practise. It is in line with the approach taken by the Society for Cardiothoracic Surgery [10]. A sensitivity analysis to assess the suitability and potential benefit of using multiple imputations was also conducted (see Supplementary materials).

### 2.4 Model development and validation

An iterative clinical and stepwise logistic regression modelling approach was used. The statistically automated selection of independent variables was based on backward-elimination via the Akaike information criterion, aiming to optimise model fit. Clinical opinion was sought and further refinement of the candidate variable list was made, and further modelling was carried out, until we developed a model which was both clinically and statistically robust.

We examined the goodness of fit (calibration) and the ability of the model to correctly separate those who went on to have the outcome from those who did not (discrimination). We used visual inspection of calibration plots derived from calculating the Hosmer–Lemeshow statistic, as opposed to relying on P values for this statistic, which are often unreliable with large datasets [11]. For assessing discrimination we used the c-statistic, which corresponds to the area under the receiver operating characteristic curve (AUC). The higher the value of the AUC, the better the model discriminates between true positive predictions and true negative predictions. An AUC of 0.5 would indicate a model which is no better at predicting the outcome than a random coin toss. In the cardiovascular disease and outcome risk prediction literature, reported AUCs are typically in the range of 0.7 to 0.95.

To improve the calibration of the model, we also explored interaction terms. A selection of clinically plausible interactions was tested by introducing them individually into the model. Those which gave a statistically significant contribution to the model were then introduced in a forward stepwise manner with manual selection on the basis of Akaike information criterion. In the interests of maintaining parsimony, we restricted the number of interaction terms to three.

The selected model was then validated on data from 2012 (n = 76,804) and calibration and discrimination tests were performed as above.

## 3 Results

### 3.1 Model development

Of the 336,443 procedures included in the model development dataset from 2007 to 2011, a total of 5722 patients died within 30 days of their procedure (1.7%). Table 3 displays the percentage of patients with each risk factor who were either alive or dead within 30 days; all risk factors except previous myocardial infarction were found to be statistically significantly associated with the outcome using a threshold of P < 0.05. In particular, the crude 30-day mortality in patients who experienced cardiogenic shock was 29.3% compared with 1.3% in those who did not; 7.2% of patients with creatinine > 200 μmol/l and 5.7% of patients on dialysis died compared to 1.6% with no renal impairment. Mortality was higher in patients whose procedure was classified as emergency (4.8%) or salvage (17.1%) than those whose procedure was elective (0.4%) or urgent (1.3%); salvage procedures being those undertaken in the context of a patient being resuscitated en route to the catheter laboratory. Similarly, mortality was higher in patients with an acute primary PCI (4.9%) or acute non-primary PCI indication (1.5%) than a stable clinical indication (0.4%).

The final model is shown in Table 4. There were nine independent risk factors in the model, all with a statistically highly significant (P < 0.001) contribution: mean-centred age, female gender, diabetes, previous myocardial infarction, renal disease, history of cerebrovascular event, clinical indication/urgency, and cardiogenic shock. Cardiogenic shock provided the largest categorical variable coefficient in the model of 3.82 (95% CI 3.43 to 4.21); this means risk of mortality is higher when that risk factor is present. Clinical indication/urgency was also weighted heavily in the model with a coefficient of 2.53 (95% CI 2.38 to 2.68) for the highest risk category (group 5) as were dialysis (coefficient 1.13, 95% CI 0.94 to 1.32) and creatinine > 200 μmol/l (coefficient 1.00, 95% CI 0.87 to 1.13).

There were three interaction terms introduced to improve the calibration of the model: clinical indication and cardiogenic shock, centred age and cardiogenic shock and centred age and diabetes. The negative coefficients associated with these interactions effectively correct for over estimation of risk in patients who have both risk factors in the interaction pair. The quadratic term for age did not improve the model so it was not included.

Risk prediction is carried out by summing constant and all coefficients where a risk factor is present to derive the log odds. 64.8 is subtracted from age before multiplying by coefficient. Interaction coefficients are used when both interacting features are present.

For example, a 75-year old male with diabetes, in urgency group 2 would have log-odds = − 6.089 + 0.071*(75–64.8) + 0.524 + 1.004 + (− 0.016) × (75–64.8) = − 4.000. This can be converted to a probability: P = 100/(1 + exp.(−(− 4.000))) = 1.80%.

### 3.2 Model validation

The validation data from 2012 comprised 75,234 procedures with a 30 day mortality of 2.09%. The validation cohort was similar to the development cohort in terms of patient characteristics though patients in the validation cohort were on average slightly older (mean age alive = 64.9, mean age dead = 74.3) and a far higher percentage of the patients who died within 30 days had been classified as salvage procedures (36.2% compared with 17.1% in the development cohort).

**Fig. 2:**
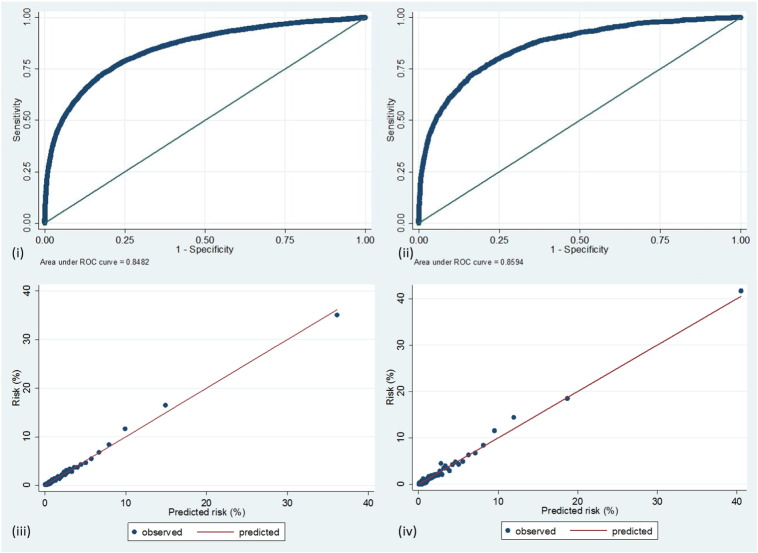
Illustrations of model discrimination and calibration. (i) Receiver operating characteristic curve of model on development data (2007–2011), (ii) receiver operating characteristic curve of model when applied to validation data (2012), (iii) calibration plot 100 quantiles) for model in development, and (iv) calibration plot (100 quantiles) for model on validation.

Fig. 2 illustrates the calibration and discrimination of the model in both development and on validation. In development, the model discrimination was good, represented by an AUC of 0.848. On validation the discrimination was maintained at a similar level, with an AUC of 0.859. This represents discriminative ability at the upper end of the range of models in this clinical domain.

Calibration plots at a range of quantile thresholds indicated a good model fit with the development data. Fig. 2(iii)shows the calibration plot for 100 risk strata. With the development cohort data the calibration plots were slightly less good, but still acceptable: Fig. 2(iv). To assess the validity of using a single model in spite of a wide range of predicted risk, we assessed its calibration and goodness of fit in the subset of patients being treated for ST elevated myocardial infarction by primary PCI and also on the subset being treated for stable angina and non-ST elevated myocardial infarction. In both subsets it performed well with AUCs of 0.822 and 0.818 respectively.

### 3.3 Sensitivity analysis

We examined different approaches to handling missing data. Complete case analysis was performed by examining the performance of the above model only in people who had information available for all the included variables (see Supplementary materials). Of the development cohort, 264,053 (78.5%) of people fulfilled this criterion. The model coefficients and intercept were broadly similar. There was an increase in the coefficient for indication-urgency group 5 but a decrease in the coefficient for cardiogenic shock. The area under the ROC curve for the complete case model was 0.789. This drop in model discrimination may lend support for the inclusion of non-complete cases under an imputation framework.

Multiple imputation was conducted as a sensitivity analysis to see if there was any additional benefit in using this approach. Overall the model coefficients remained broadly consistent under multiple imputation, though the coefficient for cardiogenic shock decreased from 3.82 to 3.73 (with a corresponding drop in odds ratio of 45.47 to 41.76). There was no difference in the AUC between ‘missing assumed absent’ and multiple imputation, as assessed using either the training or validation datasets, nor did calibration plots noticeably differ.

Inclusion of LVEF as a variable in the model under different missing data handling frameworks identified that worsening LVEF was associated with a higher odds of 30 day mortality. However, the estimated size of the effect was sensitive to the method used. When the model was trained only on cases where LVEF data were present, ‘fair’ LVEF (30–50%) was assigned a coefficient of 0.84 (OR 2.31, 95% CI 2.08–2.56, P < 0.001) and ‘poor’ LVEF (< 30%) had a coefficient of 1.67 (OR 5.31, 95% CI 4.72–5.97, P < 0.001). Where multiple imputation was used, however, the estimates shrunk: fair LVEF gave 0.28 (OR 1.33, 95% CI 1.23–1.44, P < 0.001) and poor LVEF gave 0.79 (OR 2.19, 95% CI 2.02–2.39, P < 0.001). A comparison of models where LVEF was or was not included indicated only a small incremental improvement in overall model discrimination on its inclusion: in the full training set a model without LVEF had an AUC of 0.848 while a model with LVEF had an AUC of 0.852. We decided not to include LV function in our model for a number of reasons. We wanted the model to be as parsimonious as possible. The analysis above has shown only a small improvement in AUC. Our model needed to be as robust as possible in the setting of a National data collection programme, where the majority of PCI is performed in an acute setting, the likelihood of a significant improvement in data collection for LV function was low in the short term, and the variability of LV function in this setting acknowledged but poorly defined.

## 4 Discussion

### 4.1 Summary

We have created a contemporary PCI mortality model that encompasses a wide range of clinical risk, from stable elective PCI to emergency primary PCI and cardiogenic shock. It has a high degree of discrimination and is well calibrated across the risk spectrum.

The overriding aim of clinical audit is to drive up standards of care. This requires that important aspects of care are measured so they can be assessed. Robust risk adjustment underpins any clinical audit that is used to assess institutional or individual operator performance.

There are several outcomes by which the quality of PCI might be measured. While symptom relief is undoubtedly important in patients with stable angina, over 70% of patients in the UK are treated in the context of an acute coronary syndrome [7], for which mortality is the least biased and arguably the most important outcome measure. In addition, any national audit is limited by logistic and funding issues. Our experience is that the completeness and accuracy of self-reported outcomes vary considerably. Our pragmatic solution was to use the Office for National Statistics tracked mortality as an outcome because it is independent of local data collection heterogeneity.

### 4.2 Comparison with other models

The model presented here represents a tool which better reflects contemporary UK clinical practise than existing published models. The previous UK NWQIP model [5] reflected practise before the era of widespread uptake of primary PCI, was found to be poorly calibrated in the assessment of contemporary procedures and therefore a new model was required. The new BCIS model does demonstrate some consistency with the previous model, despite the differences in outcome and patient population: in both models cardiogenic shock and degree of urgency of the procedure are heavily weighted. There are, however, several differences. We have opted to consider age as a continuous variable so as to introduce finer granularity of risk assessment in this variable. The BCIS model now includes a measure of renal function, while omitting variables relating to the lesions treated. In the latter case this was due to a priori decision to exclude variables relating to the peri-procedural period; this is pertinent to the model's intended use for appraisal of operator performance, as such variables may be influenced by operator’s decisions. In keeping with the NWQIP model, we have opted to retain the regression model weights rather than derive an integer-based score, as the latter is an anachronism of pre-computer clinical prediction. We include a measure of renal function, even though this may not be known at the time of emergency procedures as we wanted to restrict the model to risk factors present prior to procedure, whether known or not at the time of treatment. This is appropriate, given that the primary intended purpose of the model is service audit, rather than aiding pre-procedural clinical decisions.

It is possible that the discrimination of this model would improve with the addition of further risk factors. Lack of sufficient data on left ventricular ejection fraction (LVEF) was a notable limitation of our data resource. The Toronto PCI in-hospital mortality score, which has an AUC of 0.96, incorporates a binary measure of LVEF, assigning an odds ratio of 1.40 to patients with LVEF of < 20% [12]. Both this model and the US National Cardiovascular Disease registry in-hospital mortality model (AUC of 0.93) [8] incorporated measures of estimated glomerular filtration rate into models, rather than using a binary creatinine threshold as we have done here. It is intended that in future updates to the BCIS model, we will consider estimated glomerular filtration rate for incorporation, as improved collection of data on these risk factors is mandated in future data collection. Left ventricular function was not included in the model as this was missing in 50.7% of cases. Additional reasons have been discussed above but inclusion did not add usefully to AUC.

### 4.3 Using risk models in clinical practise

Clinical performance measurement is moving into an era of greater openness, transparency and candour. It is no longer acceptable for measures of outcome to be used only within professional bodies. Public reporting of outcomes of individual operators is intended to drive up standards, and uncloak what has been perceived as professional secrecy. In providing patients with much more information, it aims to help a rational selection of treatment choices, and to promote a better understanding of expected outcomes. It demonstrates to the public that quality of care is being actively monitored and improved where necessary. Public reporting also encourages healthcare organisations to focus on recording and providing more complete and accurate information.

An important problem with public reporting is that it can precipitate risk-averse clinical decisions, disadvantaging the sickest patients who have the most to benefit from interventions [13], [14], [15], [16]. If a model under-predicts risk in high risk cases, then a cardiologist taking on these cases may be incorrectly assessed to be underperforming, and recognising this will be anxious to avoid treating such patients. In the UK, cardiothoracic surgeons have been reporting outcome data for some years [17]. In a recent analysis of the performance of EuroSCORE II they demonstrated that it was poorly calibrated and had weak discrimination for emergency cardiac surgery [18]. As a result such cases are currently excluded from reports. It is therefore critically important that the model we have created performs well at extremes of risk. However, in the setting of out of hospital cardiac arrest no satisfactory risk model currently exists, and to reduce the potential harm from risk-averse clinical behaviour over patients presenting in this way, we decided to exclude them both from this model, and from public reporting of PCI outcomes in the UK for the time being [9].

### 4.4 Limitations

Although the BCIS audit programme applies to all UK procedures, the model was fit using only data from England and Wales, since record linkage via the Office for National Statistics is available only for these patients. This does, however, present opportunity for validation studies in Scotland and Northern Ireland; these regions may have different patterns of risk factors and mortality, adding useful heterogeneity to the extended validation of this model. Further temporal validation should be conducted in future years to ensure the performance of the model remains acceptable, with recalibration performed as necessary [19], [20].

Our practise of replacing missing categorical values with null values may lead to biased estimates of individual patient risk. This will cause bias if some centres or operators are systematically under-reporting the presence of risk factors. However, we found no benefit to be had from conducting multiple imputation, and it may be more productive to focus efforts on reducing missing data at source in future years of the audit. Given the additional complexity of applying the model in a multiple imputation framework, and the fact that data may be missing in patterns other than random, our approach is appropriate at this time.

Due to limitations imposed by the data sharing framework, we did not have access to un-pseudonymised procedure date. It was therefore not possible to identify redo procedures (i.e. those which were a second attempt), which may have led to some double counting of procedures or potential underestimation of the mortality burden of the index procedure in cases where a patient died following a redo procedure. We hope that the establishment of “safe havens” for analysis by national organisations such as the Farr Institute will enable such analyses in the future, better serving patients with proportionate governance of their information [21].

### 4.5 Future research

We intend to validate the model further, and monitor the calibration and discrimination performance over time, updating as necessary. Future updates of the model will likely incorporate established or emerging risk factors that we were not able to include in this version.

In addition, we are investigating dynamic modelling approaches that will run continuous statistical surveillance of model performance and prompt the clinical audit team over possible calibration drift and potential structural deterioration of the model. Where factors such as renal function are under-represented due to data quality they can be kept on watch for fuller inclusion as the data are better collected. In addition, there is a need to study the effects of different forms of audit feedback on data collection and quality.

### 4.6 Conclusions

We have generated a new parsimonious and contemporary model for predicting risk of 30-day mortality in patients undergoing PCI in the UK. It shows good discrimination and calibration across a wide spectrum of risk. It takes into account the marked changes to the performance of PCI in recent years and is independent of variations in completeness of adverse event reporting and therefore is a more appropriate choice of model for the BCIS national audit programme.

## Disclosures

The authors report no relationships that could be construed as a conflict of interest.

## Figures and Tables

**Table 1:** Groupings of BCIS field entries for clinical indication for procedure, to give a five-group classification of procedural type and urgency.

Group	Indication/urgency
1	“Stable — angina” OR “Stable — coronary/LV anatomy” OR “Staged procedure” OR “Hybrid procedure”
2	“ACS — UA, NSTEMI or convalescent STEMI” AND “urgent”
3	“ACS — UA, NSTEMI or convalescent STEMI” AND “emergency” OR “ACS — UA, NSTEMI or convalescent STEMI” AND “salvage”
4	“ACS — primary PCI for STEMI (no lysis)” OR “ACS — facilitated PCI for STEMI (lysis + PCI)” OR “Acute or sub-acute PCI thrombosis” OR “Bail out following acute complication of diagnostic cardiac catheterisation”
5	“ACS — rescue PCI for STEMI (failed lysis)” OR “ACS — PCI for re-infarction (no lysis)” OR “ACS — rescue PCI for re-infarction (failed lysis)”

ACS = acute coronary syndrome; LV = Left ventricular; UA = unstable angina; NSTEMI = non-ST elevated myocardial infarction; STEMI = ST elevated myocardial infarction.

**Table 2:** Frequency and % of missing data for each variable included in the model.

	Training data (2007–2011); n = 336,433		Validation data (2012); n = 76,804	
Variable	n missing	% missing	n missing	% missing
Age at procedure	166	0.05	9	0.01
Gender	754	0.22	90	0.12
Diabetes	13,998	4.16	4144	5.40
Urgency of procedure	361	0.11	105	0.14
Previous CABG	16,716	4.97	1763	2.30
Previous MI	38,956	11.58	5702	7.42
Renal disease	18,608	5.53	3349	4.36
Indication for intervention	5831	1.73	233	0.30
History of cerebrovascular event	146	0.04	42	0.05
Cardiogenic shock	24,269	7.21	3593	4.68

**Table 3:** Patient characteristics and crude mortality rates (%) for each variable in the training and validation datasets.

		Training dataset (2007–2011)					Validation dataset (2012)				
		Alive (n = 330,711)		Dead (n = 5722)			Alive (n = 75,234)		Dead (n = 1570)		
		n	%	n	%	P	n	%	n	%	P
Gender	Male	245,884	98.52	3701	1.48	< 0.001	55,945	98.33	952	1.67	< 0.001
	Female	84,827	97.67	2021	2.33		19,289	96.90	618	3.10	
Diabetic	No	272,427	98.43	4354	1.57	< 0.001	60,847	98.13	1161	1.87	< 0.001
	Yes (diet controlled, oral Rx, insulin)	58,284	97.71	1368	2.29		14,387	97.24	409	2.76	
Urgency	Elective	139,894	99.64	501	0.36	< 0.001	27,220	99.68	88	0.32	< 0.001
	Urgent	118,416	98.75	1499	1.25		26,196	98.71	342	1.29	
	Emergency	71,834	95.22	3605	4.78		21,774	95.13	1115	4.87	
	Salvage	567	82.89	117	17.11		44	63.77	25	36.23	
Previous CABG	No	303,359	98.27	5335	1.73	< 0.001	69,169	97.91	1476	2.09	0.003
	Yes	27,352	98.60	387	1.40		6065	98.47	94	1.53	
Previous MI	No	247,273	98.32	4222	1.68	0.089	57,124	97.98	1179	2.02	< 0.001
	Yes	83,438	98.23	1500	1.77		18,110	97.89	391	2.11	
Previous PCI	No	247,462	98.19	4568	1.81	< 0.001	56,075	97.79	1270	2.21	< 0.001
	Yes	68,611	98.82	821	1.18		17,202	98.69	228	1.31	
Renal disease/dysfunction	None	323,822	98.41	5231	1.59	< 0.001	73,475	98.08	1439	1.92	< 0.001
Creatinine > 200 μmol/l	4481	92.85	345	7.15		1177	93.12	87	6.88	
Dialysis	2408	94.28	146	5.72		582	92.97	44	7.03	
Indication	Stable	142,330	99.59	587	0.41	< 0.001	27,383	99.67	90	0.33	< 0.001
	Acute, not primary	124,665	98.53	1861	1.47		27,727	98.39	453	1.61	
	Acute, primary	62,870	95.15	3208	4.85		19,929	95.17	1011	4.83	
	Other	846	92.76	66	7.24		195	92.42	16	7.58	
History of cerebrovascular event	No	318,867	98.39	5234	1.61	< 0.001	72,326	98.07	1423	1.93	< 0.001
	Yes	11,844	96.04	488	3.96		2908	95.19	147	4.81	
Cardiogenic shock	No	327,392	98.69	4345	1.31	< 0.001	74,372	98.45	1171	1.55	< 0.001
	Yes	3319	70.68	1377	29.32		862	68.36	399	31.64	
Age	Mean age at procedure	64.6 (95% CI 64.6 to 64.7)		73.3 (95% CI 73.0 to 73.6)		< 0.001	64.9 (95% CI 64.8 to 64.9)		74.3 (95% CI 73.7 to 74.8)		< 0.001
Median age at procedure	64.9 (IQR 17)		75 (IQR 16)			65 (IQR 18)		76 (IQR 16)		

P values indicate results of statistical comparisons within datasets of patients recorded alive vs dead at 30 days (Chi squared test for categorical variables, t test for age).

**Table 4:** The final logistic regression model.

	Coefficient - log odds	Odds ratio	Odds ratio Lower CI bound	Odds ratio Upper CI bound	p
Centred age	0.071	1.073	1.069	1.077	< 0.001
Female sex	0.114	1.121	1.056	1.190	< 0.001
Diabetes	0.524	1.689	1.557	1.831	< 0.001
Previous MI	0.158	1.171	1.097	1.251	< 0.001
Renal disease					
Creatinine	0.997	2.708	2.378	3.087	< 0.001
Dialysis	1.128	3.090	2.557	3.735	< 0.001
Cerebrovascular event	0.430	1.537	1.385	1.706	< 0.001
Indication-urgency					
Group 2	1.004	2.729	2.470	3.014	< 0.001
Group 3	2.114	8.283	7.126	9.629	< 0.001
Group 4	2.295	9.921	9.013	10.921	< 0.001
Group 5	2.531	12.561	10.788	14.656	< 0.001
Cardiogenic shock	3.817	45.473	30.721	67.309	< 0.001
Age-shock interaction	− 0.026	0.975	0.968	0.982	< 0.001
Indication-shock interaction					
Group 2	− 0.951	0.386	0.246	0.605	< 0.001
Group 3	− 1.226	0.294	0.185	0.465	< 0.001
Group 4	− 1.203	0.300	0.201	0.449	< 0.001
Group 5	− 1.438	0.237	0.149	0.377	< 0.001
Age-diabetes interaction	− 0.016	0.984	0.977	0.990	< 0.001
Constant/intercept	− 6.089	0.002	0.002	0.003	< 0.001

## Appendix A Supplementary data

Supplementary material 1. Evaluation of NWQIP risk score in a contemporary, UK-wide cohort.

Supplementary material 2. Sensitivity to missing data assumptions.
