# Supplementary material for: A contemporary risk model for predicting 30-day mortality following percutaneous coronary intervention in England and Wales
Source: Int J Cardiol. 2016 May 1;210:125–32. doi: 10.1016/j.ijcard.2016.02.085 (PMC4819905; doi:10.1016/j.ijcard.2016.02.085)
Supplement: Supplementary material 1. — Evaluation of NWQIP risk score in a contemporary, UK-wide cohort. [file mmc1.docx]

**Supplementary Material**

**Evaluation of NWQIP risk score in a contemporary, UK-wide cohort**

The **NWQIP score** was developed to predict post-PCI MACCE (major adverse cerebrovascular or cardiovascular events), using 9,914 patients in North West England from August 2001 to December 2003.

Log-odds of NWQIP score is

-5.4959

+ [0.7048 * age 70–79 years] + [1.0106 * age >80 years]

+ [0.4586 * female]

+ [0.8618 * cerebrovascular disease]

+ [3.2636 * cardiogenic shock]

+ [0.4788 * urgent PCI] + [1.3625 * emergent PCI]

+ [1.6502 * LMS lesion treated]

+ [0.9101 * graft lesion treated]

NWQIP has been tested in a cohort of PCI procedures undertaken in the NHS in England and Wales, between January 2007 and December 2011. Data are obtained from the BCIS registry.

| **Differences in definitions between NWQIP and BCIS registries.** | | |
| --- | --- | --- |
|  |  |  |
|  | **NQWIP registry** | **BCIS registry** |
|  |  |  |
| **MACCE - defined as in-hospital deaths, Q-wave MI, emergency CABG, and cerebrovascular accident** | | |
| In-hospital deaths | death within the  same hospital admission regardless of cause after PCI | patient died after the procedure but before discharge |
| Q wave MI | Q wave MI was defined as a new pathological Q wave with creatine kinase more than twice the laboratory upper limit of normal with increased creatine kinase MB fraction or troponin T (ignoring non-Q wave MIs because of the subjectivity involved) | CPK >x2 upper limit of normal with elevated CK-MB or Troponin and new pathological Q waves or new LBBB. *Only complete this parameter if the patient was treated in the setting of stable angina or NSTEMI*  after procedure, before discharge |
| emergency CABG surgery | the decision to send a patient to surgery because of unstable haemodynamic status, ongoing ischaemia, threatening dissection, etc, within 24 hours of receiving PCI | The complication “emergency CABG” should be used for any emergency cardiothoracic surgical procedure (whether or not this actually involves the placing of bypass grafts).  The surgery should be:  a. Cardio-thoracic (rather than for peripheral vascular complications at access sites)  b. Be prompted and indicated by a need to  • Perform emergency revascularisation to a coronary distribution that has been the subject of a PCI or attempted PCI  and/or  • To correct as an emergency a complication of PCI such as abrupt vessel closure, cardiac or vessel perforation, dissection of a thoracic great vessel etc  after procedure, before discharge |
| Cerebro-vascular accident | Cerebrovascular accident was defined as a persistent neurological deficit at the time of patient discharge | CVA (embolic, bleed, TIA/RIND).  after procedure, before discharge |
|  |  |  |
| **Risk factors as identified in the original NWQIP model** | | |
| Cardiogenic shock | blood pressure <100 mm Hg, pulse >100 beats/min, patient cool, clammy or requiring intravenous inotropes or intra-aortic balloon pump to support blood pressure | To have Cardiogenic shock, the patient must have both  a. Systemic hypotension with a systolic BP of less than or equal to 90 mmHg  b. Evidence of peripheral hypoperfusion such as a weak pulse, pallor, cool peripheries, diaphoresis (= sweating)    Patients will usually have:  a, Pulmonary oedema / congestion  b. Relative tachycardia  c. Systemic metabolic acidosis  If these features are absent then re-consider the diagnosis.  Cardiogenic shock should only be declared after correction of other abnormalities that may cause a low systemic blood pressure – for example:  a. Hypovolaemia, blood loss or vagal reaction (To declare shock the LVEDP must be >17mmHg and the RV EDP >12 mmHg)  b. Complete heart block or other substantial arrhythmia  True cardiogenic shock is rare in cases of otherwise uncomplicated inferior myocardial infarction. |
| Cerebrovascular disease | [undefined] | History of cerebrovascular event used as proxy |
|  |  |  |
| **All other risk factors were matched on definition** | | |

Any missing risk factor was treated as absent. This occurred in less than 2% of records for the NWQIP registry, but 6.7% of records in the BCIS registry.

Model performance is assessed using three measures:

Calibration - plotting predicted vs observed MACCE rates (for each score-ordered percentile).

Discrimination - analysing AUC (area under receiver operating characteristic curve) of risk score.

Bias - analysing the intercept and slope of a logistic model regressing MACCE against the NWQIP risk score.

Performance is also assessed when stratified by procedure priority (elective, urgent, emergency/salvage).

**Observed vs predicted over time**

Overall:


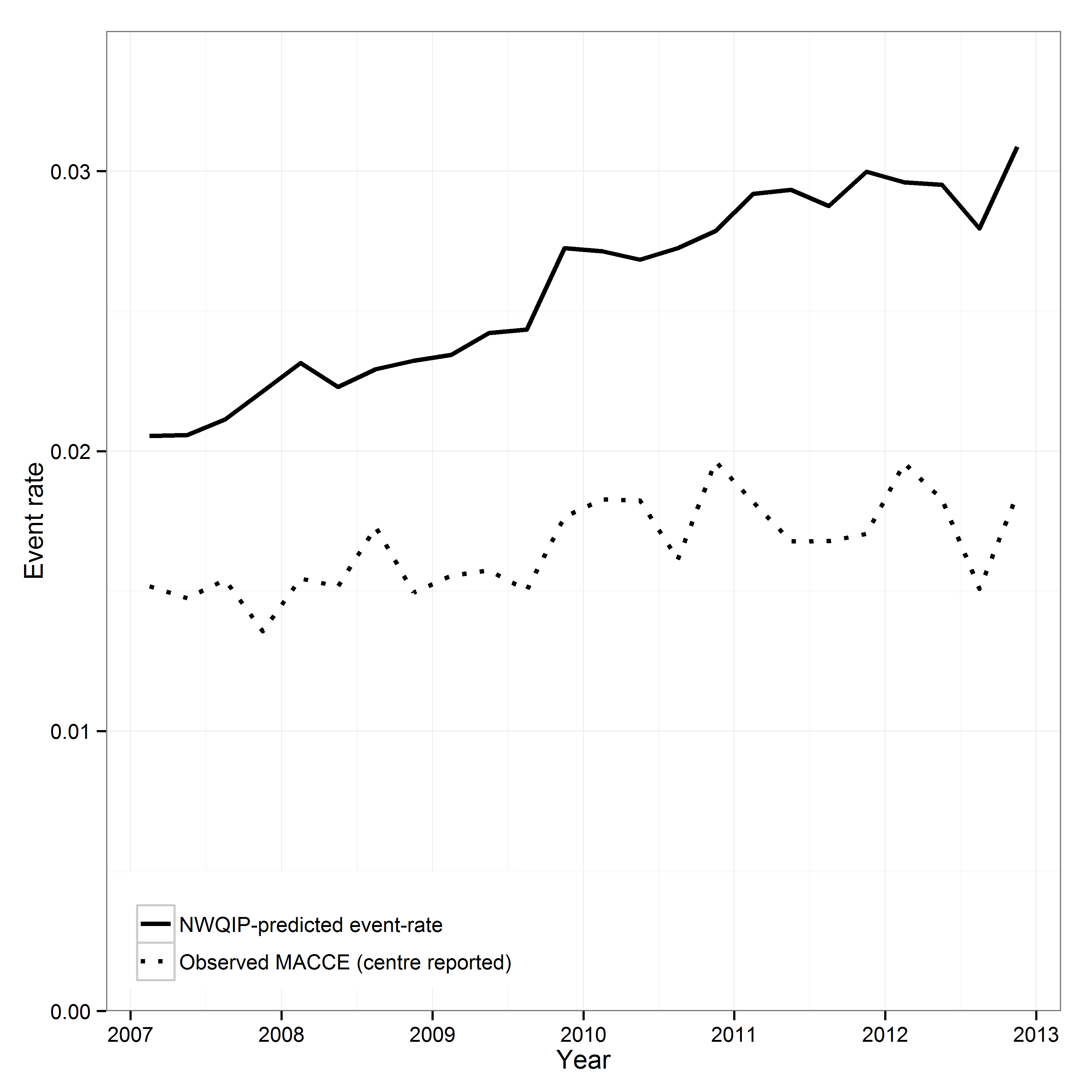


By procedure priority:


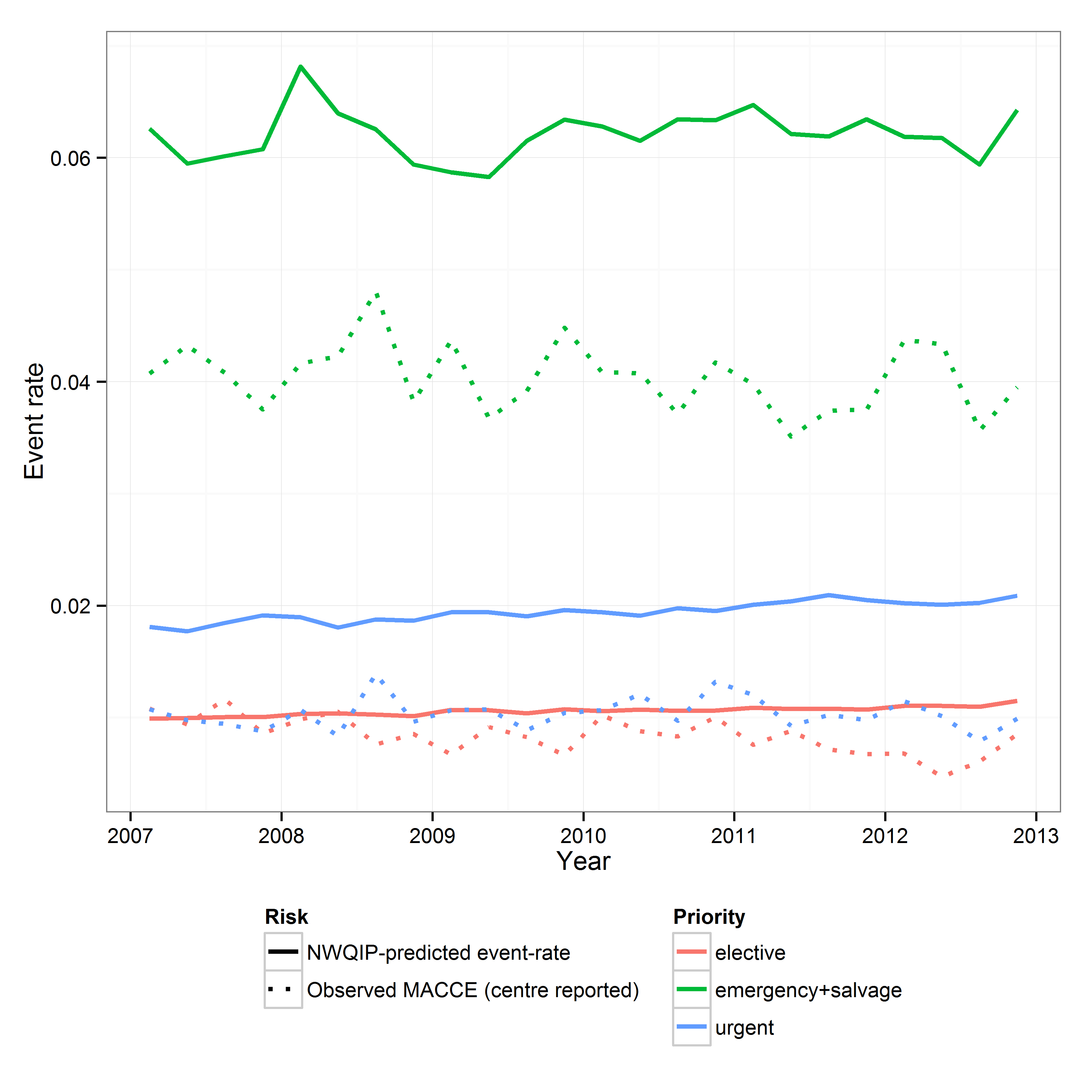


**Discrimination of NWQIP predicting MACCE in various subsets**

| **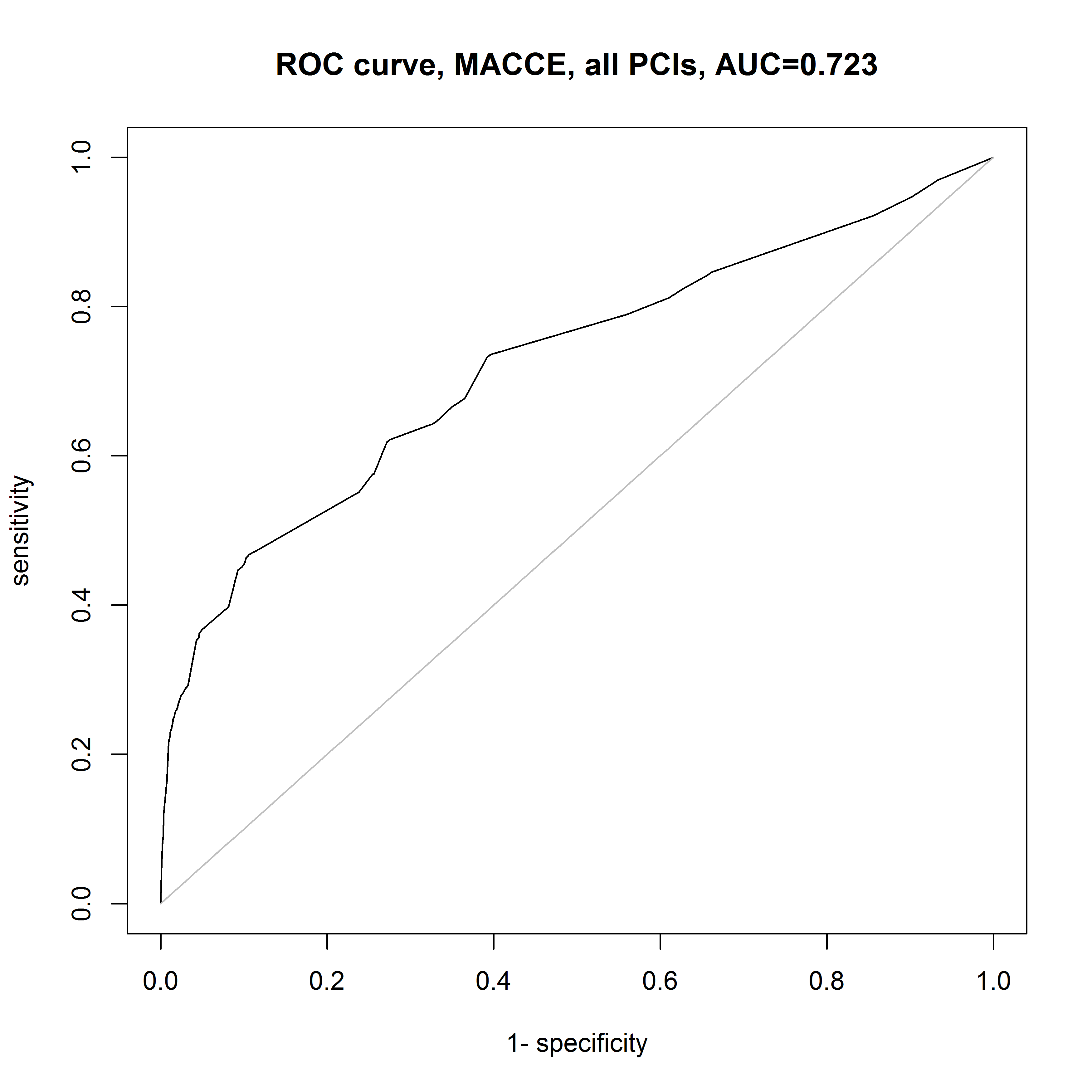** | **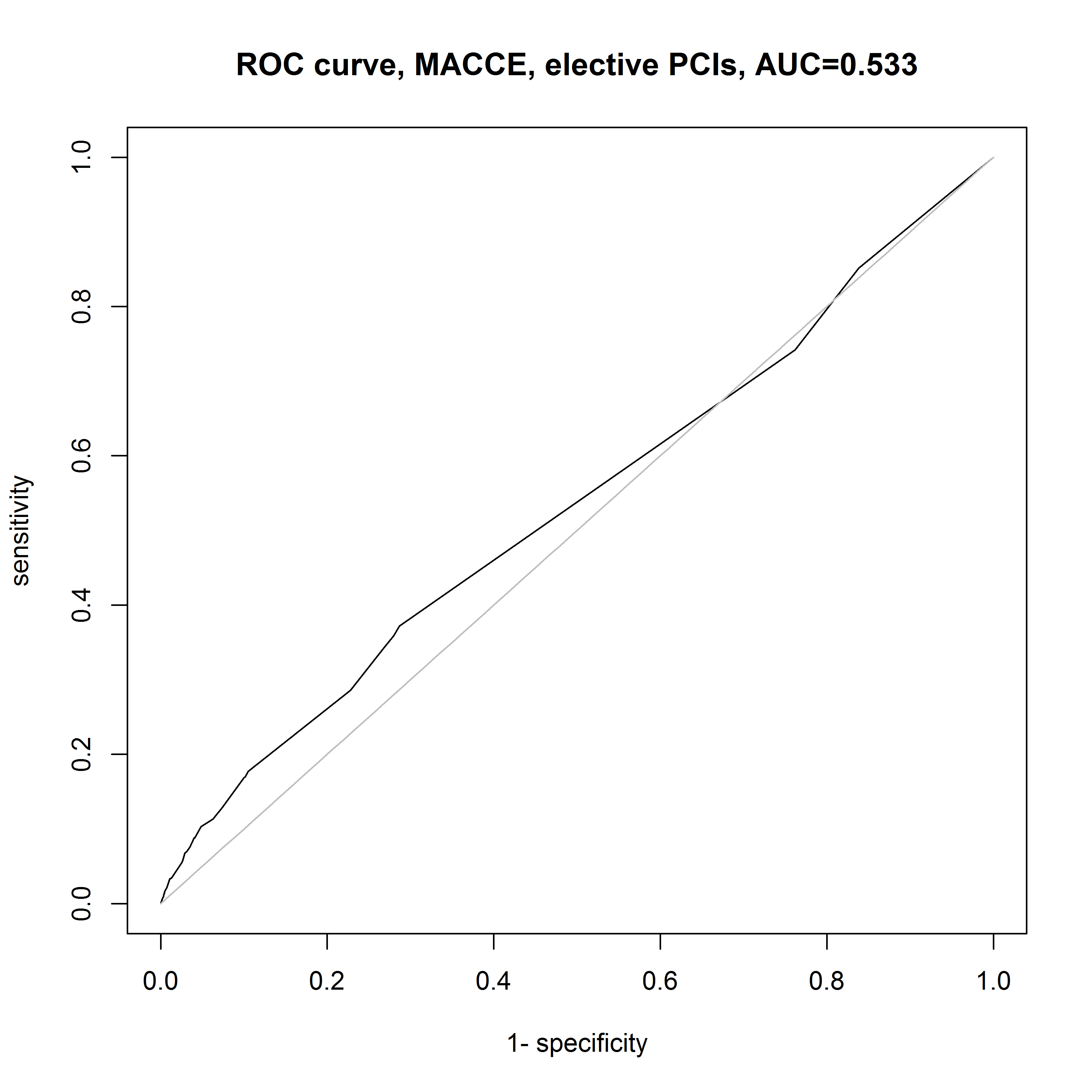** |
| --- | --- |
| **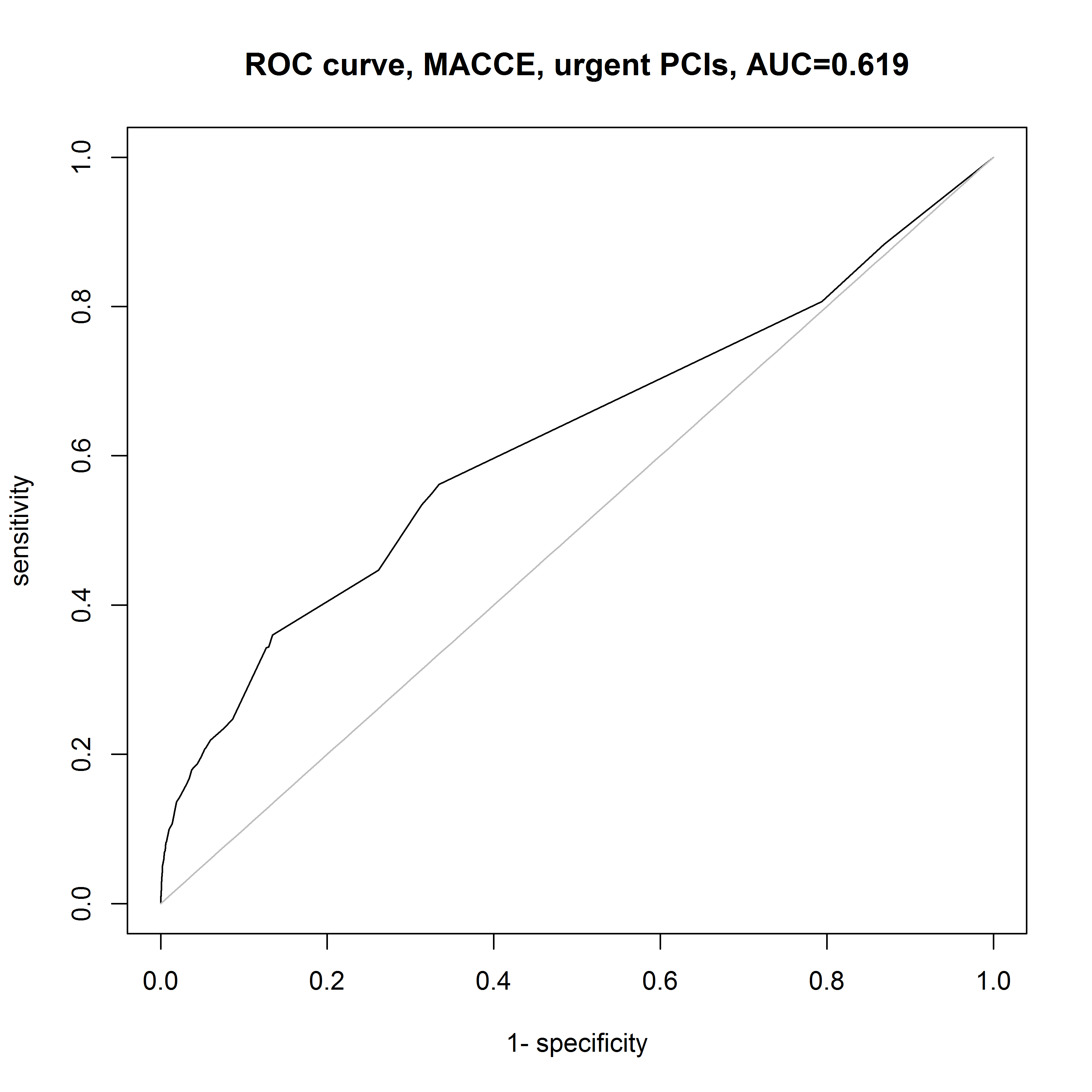** | **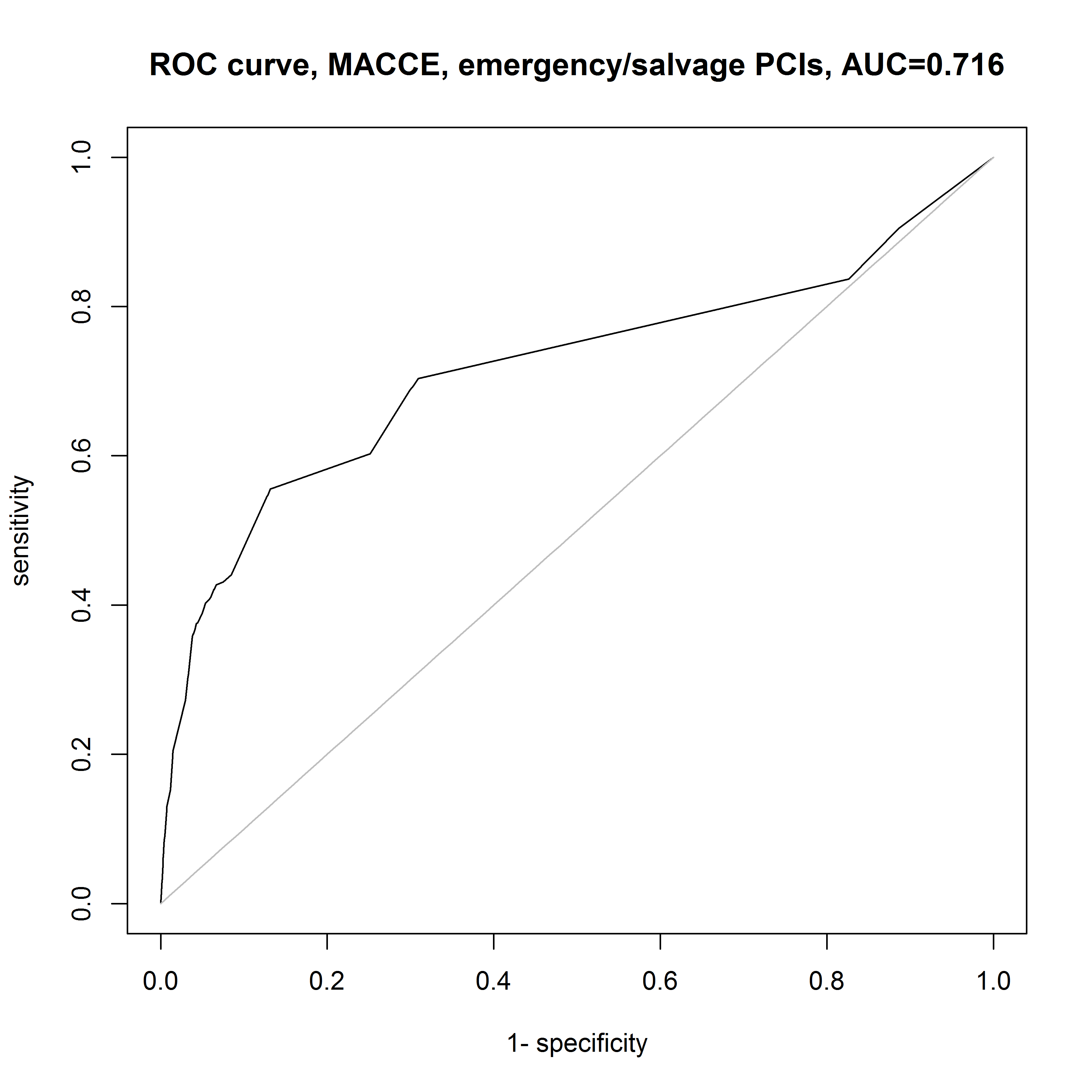** |

In particular, note the poor discrimination of the elective cases. Discrimination curve including all PCIs has the highest AUC; this reflects the importance of indication as a discriminatory predictor.

**Calibration plot**

**
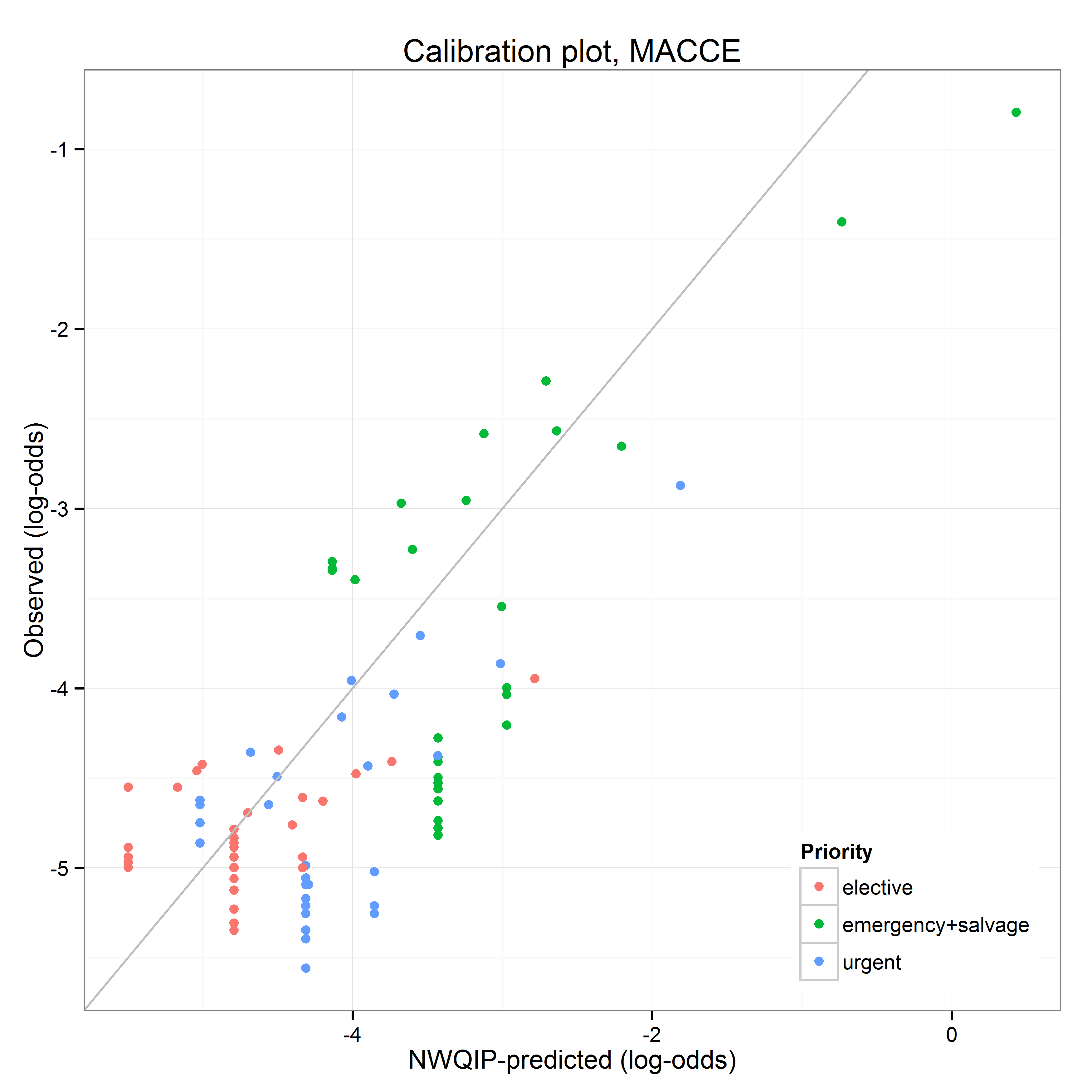
**

Overall, plots suggest over-estimation of risk of MACCE by the NWQIP model. This is most pronounced for elective and urgent PCIs.

**Bias**

The following logistic regression model

logit(probability of MACCE) = Intercept + Slope*(NWQIP logit score),

fitted on the BCIS data yields the following coefficients

Intercept = -1.19 (95% CI: -1.24, -1.14)

Slope = 0.779 (95% CI: 0.765, 0.794)

The intercept is significantly less than zero, indicating that the score over-estimates MACCE risk in the validation cohort. The slopes is significantly less than one, indicating a possible need for shrinkage in the model coefficients, i.e., the impact of risk factors are less pronounced than the model suggests.

**Conclusions**

Administrative disparities between NWQIP and BCIS registries (collection routines / definitions / missingness) may account for some of the differences upon validation. Over-estimation could be due to acknowledged under-reporting of adverse events in BCIS registry. In addition there have been changes in practice and case mix since NWQIP development.
