# Supplementary material for: A contemporary risk model for predicting 30-day mortality following percutaneous coronary intervention in England and Wales
Source: Int J Cardiol. 2016 May 1;210:125–32. doi: 10.1016/j.ijcard.2016.02.085 (PMC4819905; doi:10.1016/j.ijcard.2016.02.085)
Supplement: Supplementary material 2. — Sensitivity to missing data assumptions. [file mmc2.docx]

**Supplementary materials**

1. Sensitivity analysis: Complete case model

N=264,053 (78.5%)

AUC in complete-case validation set = 0.789

|  | **Coefficient - log odds** | **Odds ratio** | **Odds ratio Lower CI bound** | **Odds ratio Upper CI bound** | **p** |
| --- | --- | --- | --- | --- | --- |
| centred age | 0.070 | 1.073 | 1.069 | 1.076 | <0.001 |
| female sex | 0.115 | 1.122 | 1.048 | 1.201 | <0.001 |
| diabetes | 0.589 | 1.803 | 1.649 | 1.972 | <0.001 |
| previous MI | 0.206 | 1.228 | 1.142 | 1.321 | <0.001 |
| renal disease |  |  |  |  |  |
| creatinine | 1.106 | 3.023 | 2.619 | 3.489 | <0.001 |
| dialysis | 1.203 | 3.329 | 2.771 | 4.088 | <0.001 |
|  |  |  |  |  |  |
| cerebrovascular event | 0.420 | 1.521 | 1.353 | 1.710 | <0.001 |
| indication-urgency |  |  |  |  |  |
| group 2 | 1.093 | 2.984 | 2.643 | 3.370 | <0.001 |
| group 3 | 2.211 | 9.127 | 7.649 | 10.889 | <0.001 |
| group 4 | 2.377 | 10.773 | 9.567 | 12.130 | <0.001 |
| group 5 | 2.742 | 15.525 | 13.041 | 18.483 | <0.001 |
|  |  |  |  |  |  |
| cardiogenic shock | 3.473 | 32.223 | 19.083 | 54.414 | <0.001 |
|  |  |  |  |  |  |
| age-shock interaction | -0.020 | 0.981 | 0.973 | 0.988 | <0.001 |
| indication-shock interaction |  |  |  |  |  |
| group 2 | -0.588 | 0.555 | 0.312 | 0.988 | 0.045 |
| group 3 | -1.024 | 0.359 | 0.199 | 0.649 | <0.001 |
| group 4 | -0.958 | 0.383 | 0.225 | 0.654 | <0.001 |
| group 5 | -1.185 | 0.306 | 0.169 | 0.551 | <0.001 |
|  |  |  |  |  |  |
| age-diabetes interaction | -0.020 | 0.980 | 0.973 | 0.987 | <0.001 |
| Constant/intercept | -6.269 | 0.002 | 0.002 | 0.002 | <0.001 |

1. Sensitivity analysis: Multiple imputation model

| **MULTIPLE IMPUTATION MODEL** | **Coefficient - log odds** | **Odds ratio** | **Odds ratio Lower CI bound** | **Odds ratio Upper CI bound** | **p** |
| --- | --- | --- | --- | --- | --- |
| centred age | 0.071 | 1.073 | 1.070 | 1.077 | <0.001 |
| female sex | 0.114 | 1.120 | 1.056 | 1.190 | <0.001 |
| diabetes | 0.527 | 1.693 | 1.562 | 1.835 | <0.001 |
| previous MI | 0.179 | 1.179 | 1.116 | 1.281 | <0.001 |
| renal disease |  |  |  |  |  |
| creatinine | 0.980 | 2.663 | 2.340 | 3.031 | <0.001 |
| dialysis | 1.096 | 2.993 | 2.476 | 3.619 | <0.001 |
|  |  |  |  |  |  |
| cerebrovascular event | 0.431 | 1.539 | 1.387 | 1.708 | <0.001 |
| indication-urgency |  |  |  |  |  |
| group 2 | 1.009 | 2.741 | 2.482 | 3.029 | <0.001 |
| group 3 | 2.107 | 8.223 | 7.066 | 9.569 | <0.001 |
| group 4 | 2.299 | 9.968 | 9.049 | 10.979 | <0.001 |
| group 5 | 2.524 | 12.473 | 10.689 | 14.555 | <0.001 |
|  |  |  |  |  |  |
| cardiogenic shock | 3.732 | 41.760 | 28.202 | 61.836 | <0.001 |
|  |  |  |  |  |  |
| age-shock interaction | -0.025 | 0.975 | 0.969 | 0.982 | <0.001 |
| indication-shock interaction |  |  |  |  |  |
| group 2 | -0.880 | 0.415 | 0.266 | 0.648 | <0.001 |
| group 3 | -1.120 | 0.326 | 0.207 | 0.513 | <0.001 |
| group 4 | -1.110 | 0.330 | 0.221 | 0.492 | <0.001 |
| group 5 | -1.358 | 0.257 | 0.162 | 0.407 | <0.001 |
|  |  |  |  |  |  |
| age-diabetes interaction | -0.016 | 0.984 | 0.978 | 0.990 | <0.001 |
| Constant/intercept | -6.111 | 0.002 | 0.002 | 0.002 | <0.001 |
